# Study on the postoperative visual function recovery of children with concomitant exotropia based on an augmented reality plasticity model

**DOI:** 10.3389/fpsyg.2023.1025577

**Published:** 2023-09-25

**Authors:** Xiu-Fang Lv, Hui Zhong, Hao-Jiang Yang, Li He, Mei Xiong, Xiao-Ling Zhang, Li Wang, Wang Fang, Jin Wu

**Affiliations:** ^1^Department of Ophthalmology, Shenzhen Children’s Hospital, Shenzhen, China; ^2^Department of Ophthalmology, The First Affiliated Hospital of Shenzhen University (Shenzhen Second People’s Hospital), Shenzhen, China

**Keywords:** augmented reality plasticity model, children, concomitant exotropia, postoperative, visual function

## Abstract

**Objective:**

This study aimed to investigate the clinical application effect of an augmented reality (AR) plasticity model on the postoperative visual function recovery of children with concomitant exotropia.

**Methods:**

Between September 2019 and October 2021, 28 patients with concomitant exotropia who visited Shenzhen Children’s Hospital (9 male and 19 female) were enrolled in this study. The average age of the patients was 6.4 ± 1.8 years. Postoperative rehabilitation training was conducted using a personalized AR binocular visual perception plasticity model developed based on the patient’s examination results. After 1 month, 3 months, and 6 months of training, the patients returned to the hospital for examinations of perceptual eye position, static zero-order stereopsis, dynamic first-order fine stereopsis, and dynamic second-order coarse stereopsis to compare the changes in eye position control and stereovision function.

**Results:**

After 6 months of eye position training, the horizontal perception eye position of the 28 patients was significantly lower than that before training. The difference in eye position at the first and third months compared with that before training was not statistically significant (1st month: z = −2.255, *p* = 0.024 > 0.017; 3rd month: z = −2.277, *p* = 0.023 > 0.017; 6th month: z = −3.051, *p* = 0.002 < 0.017). The difference in vertical perceptual eye position after training compared with that before training was not statistically significant (1st month: z = −0.252, p = 0.801 > 0.017; 3rd month: z = −1.189, p = 0.234 > 0.017; 6th month: z = −2.225, *p* = 0.026 > 0.017). The difference in 0.8-m static zero-order stereopsis before and after training was not statistically significant (1st month: z = −2.111, p = 0.035 > 0.017; 3rd month: z = −1.097, *p* = 0.273 > 0.017; 6th month: z = −1.653, *p* = 0.098 > 0.017). The 1.5-m static zero-order stereopsis was improved after 1 month, 3 months, and 6 months of training compared with that before training (1st month: z = −3.134, *p* = 0.002 < 0.017; 3rd month: z = −2.835, *p* = 0.005 < 0.017; 6th month: z = −3.096, *p* = 0.002 < 0.017). Dynamic first-order fine stereopsis and dynamic second-order coarse stereopsis were measured in the 28 patients before and after training. Patients 1 and 18 had no dynamic first-order fine stereopsis before training, but both regained dynamic stereopsis after 1 month, 3 months, and 6 months of training. Patient 16 had no dynamic first-order fine stereopsis or dynamic second-order coarse stereopsis before training, but first-order and second-order stereopsis had been reconstructed after 1 month, 3 months, and 6 months of training.

**Conclusion:**

Concomitant exotropia surgery improved the basic problem of eye position at the ocular muscle level, but the patient’s perceptual eye position and visual function defects at the brain visual level remained. This might partly explain the poor postoperative clinical effect. The AR plasticity model can improve patients’ horizontal perceptual eye position and multi-dimensional stereoscopic function, and its clinical effect warrants further study.

## Introduction

Concomitant exotropia is a common disease in children’s ophthalmology. The incidence rate increases with age, and most cases occur in childhood. It has a progressive course, generally starting with exophoria, then continuing to intermittent exotropia, and finally developing into constant exotropia (Kaur and Gurnani, 2023). In clinical practice, surgery is an important treatment method. After surgery, the visual axis of both eyes becomes parallel, and the normal retinal correspondence is reestablished, promoting the recovery of binocular monocular function (Ha and Kim, 2016) and reducing the anxiety levels of both children and parents (Temeltürk et al., 2022). However, undercorrection is common following surgery, and the rate of regression and undercorrection are high (Yang and Hwang, 2009; Ren et al., 2015). Although strabismus surgery is necessary, it is not enough to rebuild the balanced sensory eye dominance alone (Zhou et al., 2017). Children with strabismus can only retain binocular visual axis in a parallel position for a long time if they have a higher level of binocular visual function, thereby reducing the incidence of postoperative regression and achieving the ideal therapeutic effect (Wang et al., 2022). In recent years, the application of VR to ophthalmic diseases in clinical practice has been more widely reported. Previous studies (Shibata et al., 2005; Takada et al., 2009; Jiménez-Rodríguez et al., 2023) showed that VR training could improve patients’ visual acuity, and Miao et al. (2020) demonstrated that VR could better assess the degree of eyeball deviation in patients with strabismus. Mohamed Elias et al. (2019) revealed that the ability of the eyes to adjust and focus could be affected after using VR equipment. However, reports on AR technology have only been preliminarily applied in ophthalmology teaching and surgical navigation (Li et al., 2021), and even fewer studies have been conducted on the effects of strabismus postoperative AR training. This study aimed to investigate the clinical application effect of an augmented reality (AR) plasticity model on the postoperative visual function recovery of children with concomitant exotropia.

## Data and methods

### General data

Twenty-eight patients (9 male and 19 female) who had visited our hospital with concomitant exotropia between September 2019 and October 2021 were enrolled in this study. The age of these patients ranged from 4 to 11 years, with an average age of 6.4 ± 1.8 years. All the patients received augmented reality (AR) training post-operation and were followed up in our hospital after 1 month, 3 months, and 6 months of training.

### Inclusion and exclusion criteria

(I) Inclusion criteria: (1) patients met the diagnostic criteria of concomitant exotropia, (2) patients with normal naked eye vision or corrected vision, (3) patients without organic eye diseases, and (4) patients with good cognitive skills and who were able to participate in the examinations and training.

Exclusion criteria: (1) patients with organic eye diseases, (2) corrected vision that was not up to standard, and (3) patients who had cognitive impairment and were not able to cooperate with the examinations and training.

### Examination methods

**Figure 1:**
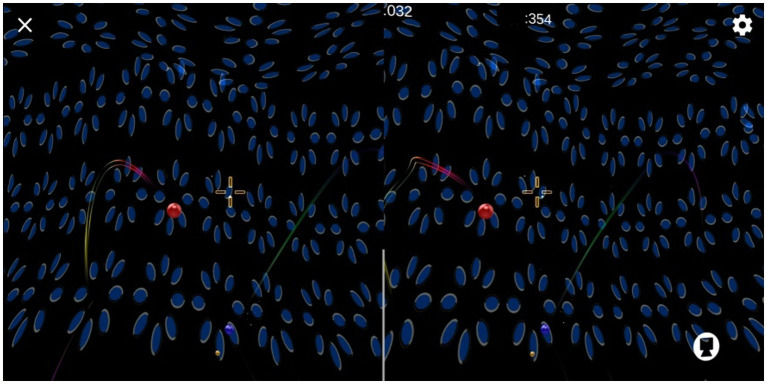
Rehabilitation training figure of AR binocular visual perception plasticity model.

1. Routine ophthalmic examination: visual examination, optometry, intraocular pressure, external eye, anterior segment, and fundus examination.

2. Instrumentation and examination system: The computer was equipped with Windows XP and LG2342p positive 3D display, with a display resolution of 1,920 × 1,080 and a refresh rate of 120 Hz. Used AR helmet (Visual bioinformatics stimulation therapy software (Yan Li et al., 2006), Galen RS20YY-JT 2005 inspection system, the National Medical and Health Appliance Engineering Technology Research Center, Guangdong, China) to train the children.

3. Examination methods: The patient wore polaroid eyeglasses in both eyes during the examination. If the patient wore corrective glasses, polaroid glasses were superimposed. The patient remained in a sitting position 0.8 m away from the display. The patients’ eyes were at the same height as the examination items. The patient operated the examination items using a mouse or keyboard.

4. Examination contents (Tan et al., 2020).

- Perceptual eye position examination: After wearing polaroid eyeglasses, the patients looked at the circle with their left eye and the plus sign with their right eye and were instructed to put the plus sign in the circle. After two attempts, the system displayed the deviation values of the horizontal and vertical eye positions.
- Static zero-order stereopsis: When the letter E appeared on the examination screen, the patient needed to judge its orientation. The level increased with three consecutive correct judgments, and there were four levels in total. The highest stereopsis level is level 4, followed by levels 3, 2, and 1, and the corresponding optical parallaxes were 100″, 200″, 300″, and 400″.
- Dynamic first-order fine stereopsis: When a moving letter E appeared on the examination screen, the patient needed to judge its orientation. The presence of dynamic first-order fine stereopsis was determined based on three consecutive correct judgments; a correct judgment scored 1 and an error scored 0.
- Dynamic second-order coarse stereopsis: The patient needed to judge whether the visual target in the figure was at a peak or trough based on a total of four judgments. When all four judgments were correct, an accuracy rate of 100% was achieved, which scored 1; otherwise, a score of 0 was given.

5. Training contents: In the binocular split vision state, the peripheral visual field was a large-scale neural reasoning stereoscopic dynamic wave (binocular parallax changed sinusoidally), with moving stereoscopic balls appearing randomly in the field of vision (the good eye signal was stationary, and the poor eye signal vibrated around the signal of the good eye) and automatic bullets constantly appearing in the collimation part of the foveal field of vision (see Figure 1). Wearing an AR helmet, the patient used head movement signals to control the collimation and break the moving three-dimensional ball in the field of vision. The duration was controlled at approximately 10 min.

During the training process, AR allows the patient to integrate real information from both eyes. The training was conducted at a distance of 80 cm and 5 m from the obstacle in relation to the far and near stereoscopic function of the patient. Based on the patient’s perceptual position in both eyes, by wearing an AR helmet and constantly rotating the head for training in all directions, the patient was able to further coordinate the relationship between eye movement and visual vestibule, thereby controlling eye position and enhancing multi-dimensional stereoscopic function.

6. Training method: Visual perception training was conducted through a personalized training program developed based on the patient’s examination results and using a family model. The patient logged into the account on their mobile phone for AR training (completed in the parental supervision mode), trained four times a day (The four training sessions were not scheduled for a fixed period of time, and patients were allowed to exercise during their free time. Usually twice in the morning and twice in the afternoon or evening), two items at a time, with 10 min for each item, and the interval between each training item was 10 min. All the training was conducted under corrected vision. The effect of plasticity AR training was reviewed every month, and the parameters of the training model were adjusted according to the results of the visual function test and short-term plasticity changes.

### Statistical methods

All data were analyzed using the SPSS22.0 statistical software package (the paired nonparametric test or Wilcoxon signed rank sum test). Because only three tests were conducted, to avoid statistical errors, the inspection level was adjusted to α’ = 0.017 instead of α = 0.05; thus, α/3 = 0.05 ÷ 3 = 0.017.

## Results

### Statistics of patient demographics

A total of 28 patients aged 4 to 11 years were included in this study, with 9 boys (32.1%) and 19 girls (67.9%). The average age was 6.4 ± 1.8 years, as detailed in Table 1.

**Table 1:** Statistics of demography and perceptual eye position of 28 cases of patients.

	Before training	After training for 1 month	After training for 3 month	After training for 6 month
Number	28	28	28	28
Age	6.4 ± 1.8y	6.4 ± 1.8y	6.4 ± 1.8y	6.4 ± 1.8y
Horizontal PEP pixels	141.0 (49.5,277.5)	101.5 (42.0,214.25)	75.5 (36.75,145.75)	46.0 (30,157.5)
Vertical PEP pixels	14.5 (8.25,24.75)	10.5 (5.25,24.0)	6.5 (4.25,22.25)	8.0 (5.0,14.0)

### Comparison of horizontal perceptual eye position and vertical perceptual eye position before and after training

**Figure 2:**
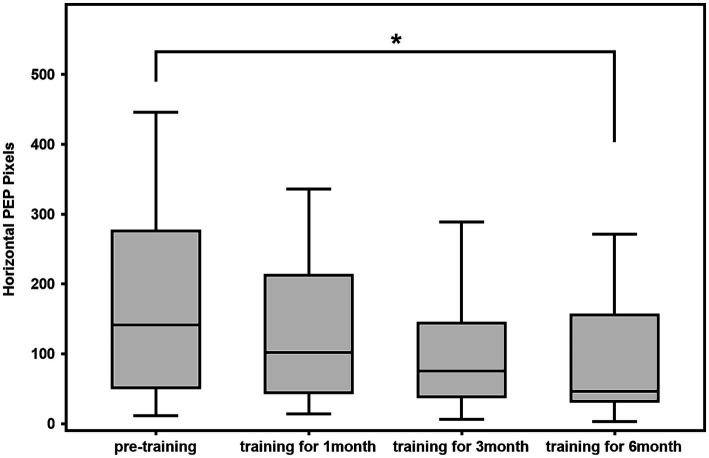
Box diagram of horizontal perceptual eye position of 28 cases of patients.

**Figure 3:**
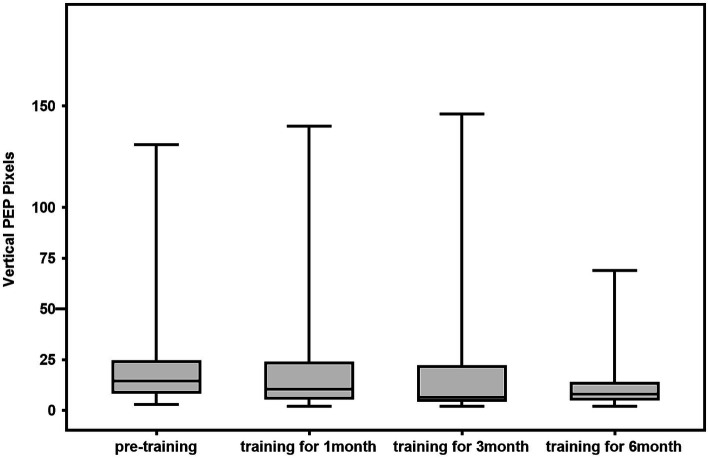
Box diagram of vertical perceptual eye position of 28 cases of patients.

The 28 patients were trained for 6 months, after which the horizontal perceptual eye position of all 28 patients was significantly lower than that before training. The difference in horizontal perceptual eye position at the first and third months compared with that before the training was not statistically significant (1^st^ month: z = −2.255, *p* = 0.024 > 0.017; 3rd month: z = −2.277, *p* = 0.023 > 0.017; 6^th^ month: z = −3.051, *p* = 0.002 < 0.017). The difference in vertical perceptual eye position after training compared with that before training was not statistically significant (1^st^ month: z = −0.252, *p* = 0.801 > 0.017; 3^rd^ month: z = −1.189, *p* = 0.234 > 0.017; 6^th^ month: z = −2.225, *p* = 0.026 > 0.017; Figures 2, 3).

### Comparison of static zero-order stereopsis before and after training

**Figure 4:**
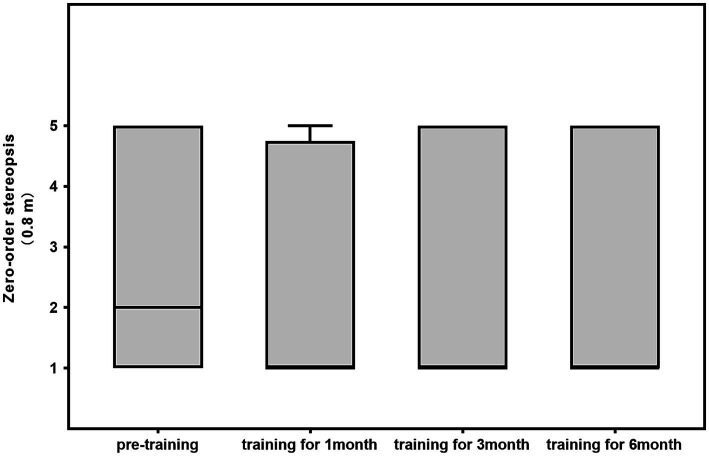
0.8-m static 0-order stereopsis of 28 cases of patients.

**Figure 5:**
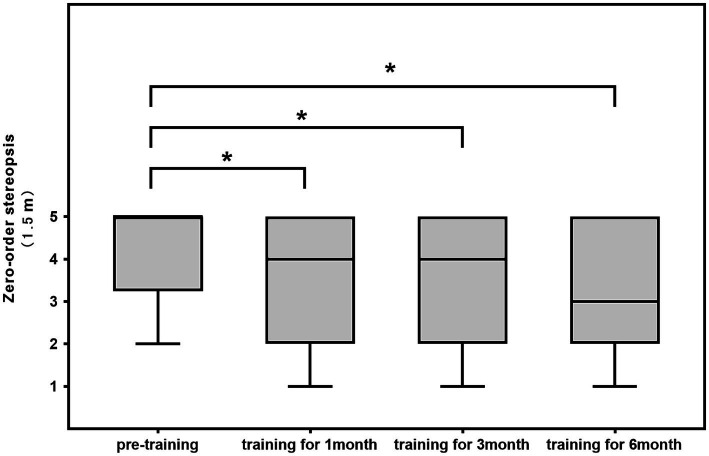
1.5-m static 0-order stereopsis of 28 cases of patients.

The difference in 0.8-m static zero-order stereopsis before and after training was not statistically significant (1st month: z = −2.111, *p* = 0.035 > 0.017; 3rd month: z = −1.097, *p* = 0.273 > 0.017; 6th month: z = −1.653, *p* = 0.098 > 0.017). A comparison of the differences in 1.5-m static first-order stereopsis before and after training revealed that it improved after 1 month, 3 months, and 6 months of training (1st month: z = −3.134, *p* = 0.002 < 0.017; 3rd month: z = −2.835, *p* = 0.005 < 0.017; 6th month: z = −3.096, *p* = 0.002 < 0.017; Table 2; Figures 4, 5).

**Table 2:** Comparison of static 0-order stereopsis of 28 cases of patients before and after training.

Number	Gender	Age	S0 (0.8 m) -before	S0 (0.8 m) -after 1 month	S0 (0.8 m) -after 3 month	S0 (0.8) -after 6 month	S0 (1.5 m) -before	S0 (1.5 m) -after 1 month	S0 (1.5 m) -after 3 month	S0 (1.5 m) -after 6 month
1	F	5y	5	5	4	5	5	5	5	5
2	F	5y	1	1	1	1	2	2	2	2
3	F	5y	2	3	5	5	5	5	5	5
4	F	7y	1	1	1	1	5	4	4	2
5	F	7y	5	5	5	5	5	5	5	5
6	F	4y	2	1	1	1	5	2	2	1
7	F	4y	5	5	5	5	5	5	5	5
8	F	11y	1	1	1	1	3	2	2	2
9	F	7y	2	1	1	1	5	3	5	5
10	F	5y	1	1	1	1	2	2	2	1
11	F	8y	1	1	1	1	4	4	4	2
12	M	5y	1	1	1	1	2	1	1	1
13	M	9y	1	1	1	1	5	5	5	2
14	F	8y	1	1	1	1	2	1	1	1
15	M	7y	1	1	1	1	3	1	1	2
16	F	4y	5	4	2	2	5	5	5	5
17	F	6y	1	1	1	1	4	4	3	4
18	M	6y	5	2	1	1	5	5	2	2
19	M	4y	5	5	5	5	5	5	5	5
20	F	6y	5	5	5	5	5	5	5	5
21	M	9y	5	5	5	5	5	5	5	5
22	F	6y	5	5	5	5	5	5	5	5
23	F	7y	2	1	1	1	5	4	5	5
24	M	8y	1	1	1	1	2	2	2	2
25	M	8y	1	1	1	1	4	3	2	2
26	F	4y	2	1	1	1	5	3	3	4
27	M	8y	1	1	5	1	4	1	5	5
28	F	6y	2	1	1	1	5	4	3	2

F: female; M: male, 1 represents static 0-order stereopsis is regarded as level 1; 2 represents level 2; 3 represents level 3; 4 represents level 4; 5 represents no static 0-order stereopsis.

### Results of dynamic first-order fine stereopsis and dynamic second-order coarse stereopsis before and after training in 28 patients

Dynamic first-order fine stereopsis and dynamic second-order coarse stereopsis were tested in the 28 patients before and after training. Patients 1 and 18 had no dynamic first-order fine stereopsis before training but had regained dynamic stereopsis after 1 month, 3 months, and 6 months of training. Patient 16 had no dynamic first-order fine stereopsis or dynamic second-order coarse stereopsis before training, and the examinations revealed that first-order and second-order stereopsis had been reconstructed after 1 month, 3 months, and 6 months of training (Table 3).

**Table 3:** Results of dynamic first-order fine stereopsis and dynamic second-order coarse stereopsis before and after training in 28 patients.

Number	Gender	Age	S1-before	S1 -after 1 month	S1 -after 3 month		S1 -after 6 month	S2-before	S2 -after 1 month	S2 -after 3 month	S2 -after 6 month
1	F	5y	0	1	1		1	1	1	1	1
2	F	5y	1	1	1		1	1	1	1	1
3	F	5y	1	1	1		1	1	1	1	1
4	F	7y	1	1	1		1	1	1	1	1
5	F	7y	0	0	0		0	0	0	0	0
6	F	4y	1	1	1		1	1	1	1	1
7	F	4y	0	0	0		0	1	1	1	1
8	F	11y	1	1	1		1	1	1	1	1
9	F	7y	1	1	1		1	1	1	1	1
10	F	5y	1	1	1		1	1	1	1	1
11	F	8y	1	1	1		1	1	1	1	1
12	M	5y	1	1	1		1	1	1	1	1
13	M	9y	1	1	1		1	1	1	1	1
14	F	8y	1	1	1		1	1	1	1	1
15	M	7y	1	1	1		1	1	1	1	1
16	F	4y	0	1	1		1	0	1	1	1
17	F	6y	1	1	1		1	1	1	1	1
18	M	6y	0	1	1		1	1	1	1	1
19	M	4y	0	0	0		0	0	0	0	0
20	F	6y	1	1	1		1	1	1	1	1
21	M	9y	0	0	0		0	0	0	0	0
22	F	6y	1	1	1		1	1	1	1	1
23	F	7y	1	1	1		1	1	1	1	1
24	M	8y	1	1	1		1	1	1	1	1
25	M	8y	1	1	1		1	1	1	1	1
26	F	4y	1	1	1		1	1	1	1	1
27	M	8y	1	1	1		1	1	1	1	1
28	F	6y	1	1	1		1	1	1	1	1

F: female; M: male.

## Discussion

With the development of brain science and visual neuroscience, brain neural plasticity can be used to quickly restore neuron cell function and activate relevant visual pathways through the visual perceptual plasticity model, which is based on various engineering implementation platforms, to repair defective visual functions. AR technology is based on the design philosophy of head-mounted display devices (Baashar et al., 2023) and is a technology that integrates the virtual scene with the real environment to supplement and enhance the real world (Sutherland et al., 2019). This technology adds a certain number of virtual components and elements to a real space to make people feel as though they have entered the virtual world (Zhang et al., 2022). This technology has been applied to laparoscopic surgery, arthroplasty, spine surgery, cranial surgery, oral and maxillofacial surgery, urology, and neuroanatomy, as well as to other fields (Han et al., 2022; Lin et al., 2022; Mendez-Lopez et al., 2022; Pojskić et al., 2022; Roberts et al., 2022; Sumdani et al., 2022; Tanzer et al., 2022). With further technological innovations, AR has begun to penetrate the routine tasks of ophthalmology in the same way as virtual reality (VR). At present, the research focus of postoperative treatment strategies for concomitant strabismus is to establish normal binocular vision function and reduce the postoperative recurrence rate (Zhang et al., 2023). Surgery is the first treatment step, and the recovery of binocular visual function plays a key role in treatments (Yu et al., 2016). Postoperative visual exercise can promote the recovery and establishment of stereopsis (Chen et al., 2023). By constructing a virtual–real fusion training scene for patients by wearing an AR helmet, the patients can see the rendered virtual environment through the monitor, gradually restoring the cognitive ability of the brain through visual feedback and promoting the recovery of binocular vision (Sutherland et al., 2019). We know that patients with concomitant exotropia, especially those with intermittent exotropia, have different stereopsis in near and far vision. In other countries, long-distance stereopsis outside is often used as a surgical indication, and this problem can be solved by using AR plasticity models.

### Recovery of binocular visual perceptual position after concomitant exotropia surgery

In recent years, with the development of brain vision science, we have a greater understanding of the optic nerve center processing site, afferent and efferent channels, and how to quantify the energy map of the stereopsis model (Coubard et al., 2014). Perceptual eye position is the expression of the separation control of eye position by the visual center in the state of binocular dichotomy. Perceptual eye position abnormality might indicate the state and degree of damage to the central perceptual function (Wu et al., 2018). Han et al. (2020) used the binocular spatial distortion biological model in the visual perception examination and treatment system for 48 patients with intermittent exotropia after surgery to conduct binocular integration function examination and reinforcement training. It was found that the patient’s perceptual eye position was significantly improved. Tan et al. (2020) conducted a 3-month two-vision visual perception training based on a unique virtual reality platform for 33 children with corrected strabismus, with the results revealing that perceptual eye position deviation was lower after training than that before training. In this study, 28 postoperative patients with concomitant exotropia underwent perceptual eye position examination before training. Personalized formulas were developed based on the results, and AR was used to conduct visual perception training. The results revealed little change in the horizontal perceptual eye position at the first and third months compared with that before training and that at the sixth-month reexamination, the differences in horizontal perceptual eye position were statistically significant. Li et al. (2019) conducted visual perception training using VR with 25 patients with intermittent exotropia. Their results also revealed no significant difference in the horizontal perceptual eye position on reexamination at the third month, and the differences were statistically significant on reexamination after 6 months. This may be related to the extra time and repetitions required to establish new neural reflexes. These results lay the groundwork for us to further explore whether long-term plasticity can develop from functional changes to the establishment of new structural changes.

### Recovery of multi-dimensional stereopsis after concomitant exotropia surgery

Stereopsis refers to the function of the eyes and visual center to perceive the orientation, distance, and depth of various objects on the spatial *x*, *y*, and *z* coordinate axes (Nityananda and Read, 2017). Stereopsis and binocular vision are crucial for unique depth perception under normal daily visual conditions (Singh et al., 2022). Meng et al. (2021) divided 133 postoperative patients with concomitant strabismus into observation group, traditional binocular vision training group and visual perception training group. The patients in the traditional binocular vision training group and the visual perception training group received the corresponding training for visual training. The results revealed that after 3 and 6 months of training, the differences in the improvement of near stereopsis were not statistically significant, but long-distance stereopsis had improved significantly 3 and 6 months after the surgery. These results are consistent with the results of the present study. This may be related to the late onset of exotropia in patients and the better postoperative preservation of near stereopsis in most patients with strabismus. Lu (1999) also studied 132 patients with intermittent exotropia, with the results revealing that intermittent exotropia was characterized by the preservation of near stereovision and loss of remote stereovision. Yan et al. (2019) included 36 adult patients with concomitant strabismus, who underwent short-term visual plasticity training after surgery. Specific operations were as follows: The patient started binocular visual perception training on the first day after surgery, training 30 min/ time, rest 10 min in the middle, training 1–2 times/day, the interval of two training hours is more than 2 h, a total of 5 training times. With the results revealing that after five training sessions, dynamic first-order fine stereopsis and dynamic second-order coarse stereopsis were improved compared with those before the training. In this study, of the 28 postoperative patients with concomitant exotropia surgery, seven had defects in dynamic first-order fine stereopsis and dynamic second-order coarse stereopsis postoperation, with improvements in three of these patients after training, suggesting that visual training can improve dynamic first-order fine stereopsis and dynamic second-order coarse stereopsis. A study concluded that, in addition to the classifications of short-distance and long-distance stereopsis, humans also have three different levels of stereopsis (Hess and Wilcox, 2008). These are the zero-order stereo disparity in the v1 area, the first-order stereo disparity in the linear change area, and the second-order stereo disparity in the curved change area (Coubard et al., 2014). After the detection of stereopsis in different channels, the energy map of the stereopsis and threshold of the corresponding model were identified (Ding and Levi, 2010). Through personalized training for multi-dimensional stereopsis, recovery from the higher visual cortex to the lower visual cortex can be achieved; for example, the zero-order fine stereopsis function was repaired by the residual second-order coarse stereopsis, establishing new plasticity repair ideas and methods for some patients with clinical “stereo blindness” and severe partial visual function defects.

### Limitations of this study

Because of the long follow-up time in this study, many patients missed follow-up for objective and subjective reasons, reducing the sample size of the study and its accuracy. Therefore, in a future study, we hope to increase the sample size and extend the research period. Furthermore, this study is a self-controlled study without a non-training control group, which limited extrapolation.

## Conclusion and prospect

Concomitant exotropia surgery improved the basic problem of eye position at the ocular muscle level, but the patient’s perceptual eye position and visual function defects at the brain visual level remained. This might partly explain the poor postoperative clinical effect. The AR plasticity model can improve patients’ horizontal perceptual eye position and multi-dimensional stereoscopic function, and its clinical effect warrants further study. At present, the application of AR technology has been closely integrated with clinical practice in many aspects. In the future, we can try to combine AR technology with artificial intelligence to improve the diagnostic efficiency of eye diseases, including exotropia. At the same time, AR technology can be introduced during surgery to provide real-time navigation and assistance to doctors, avoid vital organs and nerves during surgery, and reduce the occurrence of surgical risks and complications. At the same time, AR technology can also help patients better understand the preoperative, intraoperative and postoperative treatment process, so that they have a fuller understanding of the treatment and shorten the postoperative recovery time. The application of AR technology can also enable patients to conduct corresponding training at home, without the need to regularly go to the hospital for treatment, which is convenient for patients.

## Data availability statement

The original contributions presented in the study are included in the article/supplementary material, further inquiries can be directed to the corresponding author.

## Ethics statement

The studies involving humans were approved by the study was conducted in accordance with the Declaration of Helsinki (as was revised in 2013). The study was approved by Ethics Committee of the Shenzhen Children’s Hospital (No. 7412281). The studies were conducted in accordance with the local legislation and institutional requirements. Written informed consent for participation in this study was provided by the participants' legal guardians/next of kin.

## Author contributions

X-FL: conception and design of the research, analysis and interpretation of the data, and writing of the manuscript. LH, LW, WF, JW: acquisition of data. H-JY and HZ: statistical analysis. X-FL and X-LZ: obtaining financing. X-FL and H-JY: critical revision of the manuscript for intellectual content. All authors contributed to the article and approved the submitted version.

## Funding

This study was supported by the Shenzhen Children’s Hospital Foundation of China (No. Ynkt2020zz07).

## Conflict of interest

The authors declare that the research was conducted in the absence of any commercial or financial relationships that could be construed as a potential conflict of interest.

## Publisher’s note

All claims expressed in this article are solely those of the authors and do not necessarily represent those of their affiliated organizations, or those of the publisher, the editors and the reviewers. Any product that may be evaluated in this article, or claim that may be made by its manufacturer, is not guaranteed or endorsed by the publisher.
